# Development of a CRISPRi Human Retinal Pigmented Epithelium Model for Functional Study of Age-Related Macular Degeneration Genes

**DOI:** 10.3390/ijms24043417

**Published:** 2023-02-08

**Authors:** Jiang-Hui Wang, Daniel Urrutia-Cabrera, Jarmon G. Lees, Santiago Mesa Mora, Tu Nguyen, Sandy S. C. Hung, Alex W. Hewitt, Shiang Y. Lim, Thomas L. Edwards, Raymond C. B. Wong

**Affiliations:** 1 Centre for Eye Research Australia, Royal Victorian Eye and Ear Hospital, Melbourne, VIC 3002, Australia; 2 Ophthalmology, Department of Surgery, University of Melbourne, Melbourne, VIC 3010, Australia; 3 O’Brien Institute Department, St Vincent’s Institute of Medical Research, Fitzroy, VIC 3065, Australia; 4 Departments of Surgery and Medicine, University of Melbourne, Melbourne, VIC 3010, Australia; 5 Menzies Institute for Medical Research, School of Medicine, University of Tasmania, Hobart, TAS 7000, Australia

**Keywords:** age-related macular degeneration, retinal pigmented epithelium, CRISPR interference, retinal degeneration, *TMEM97*

## Abstract

Age-related macular degeneration (AMD) is a blinding disease characterised by dysfunction of the retinal pigmented epithelium (RPE) which culminates in disruption or loss of the neurosensory retina. Genome-wide association studies have identified >60 genetic risk factors for AMD; however, the expression profile and functional role of many of these genes remain elusive in human RPE. To facilitate functional studies of AMD-associated genes, we developed a human RPE model with integrated CRISPR interference (CRISPRi) for gene repression by generating a stable ARPE19 cell line expressing dCas9-KRAB. We performed transcriptomic analysis of the human retina to prioritise AMD-associated genes and selected *TMEM97* as a candidate gene for knockdown study. Using specific sgRNAs, we showed that knockdown of *TMEM97* in ARPE19 reduced reactive oxygen species (ROS) levels and exerted a protective effect against oxidative stress-induced cell death. This work provides the first functional study of *TMEM97* in RPE and supports a potential role of *TMEM97* in AMD pathobiology. Our study highlights the potential for using CRISPRi to study AMD genetics, and the CRISPRi RPE platform generated here provided a useful in vitro tool for functional studies of AMD-associated genes.

## 1. Introduction

Age-related macular degeneration (AMD) is one of the leading causes of blindness in the western world, with a prevalence of ~8.7%, and is projected to cause vision loss in 288 million people worldwide by 2040 [1]. AMD is characterised by abnormalities of retinal pigment epithelium (RPE) at the macula region, leading to degeneration of the overlying photoreceptors and, subsequently, progressive loss of vision. Genetic and epidemiologic studies have greatly advanced our understanding of AMD pathology. Genome-wide association studies (GWAS) have successfully identified loci associated with genetic variants implicated with AMD risk and traits, with over 60 genetic variants being associated with AMD [2,3,4,5,6], including variants within the *APOE, CFH*, *ARMS2/HTRA1* and *TMEM97*/*VTN* loci that confer risk of developing AMD. Integration with gene expression data in expression quantitative trait loci (eQTL) studies have further improved the methods to identify AMD-associated genes; however, pinpointing the exact gene variants within the loci that confer AMD risks remain challenging, which is reflective of the complexity of AMD genetics (reviewed in [7]). Understanding the function of these AMD-associated genes in the retina, as well as their expression patterns within the major retinal cells, would be important to unravel the complex genetics of AMD; however, many AMD-associated genes in the retina remain understudied. In particular, a landmark GWAS study by Frische et al. identified that the SNP rs11080055, located within the *TMEM97* gene, is associated with AMD [2]. *TMEM97* was recently identified as the Sigma-2 receptor [8], which plays a key role in mediating Ca^2+^ and K^+^ signalling cascades in the endoplasmic reticulum and has been implicated in cancer development and a range of neurological degenerative diseases [9]. Previous studies showed that activation of Sigma-2 receptor signalling by a number of different ligands induces apoptosis, cytotoxicity, and generation of reactive oxygen species [9,10,11]. On the other hand, Sigma-2 receptor antagonists were shown to provide a protective effect following traumatic brain injury [12]. The Sigma-2 receptor signalling pathway has emerged as an attractive therapeutic target to treat several CNS degenerative diseases, such as Alzheimer’s disease and schizophrenia [13,14]. Development of new tools to facilitate functional study of AMD associated genes in RPE, such as *TMEM97*, would be important to better understand the mechanisms implicated in AMD pathobiology.

The emergence of CRISPR technology provides an efficient toolset for gene editing in the retina [15]. Moreover, a recent development has highlighted the use of a nuclease-null Cas9 coupled with transcriptional repressors to modulate endogenous gene expression levels, termed CRISPR interference (CRISPRi), which offers a robust system to study gene function [16]. Notably, CRISPRi exhibits minimal off-target effects and is capable of multiplexed gene repression [17]. Several CRISPRi sgRNA libraries are also available, which allows high-throughput genome-wide screening of gene repression [18,19]. In this study, we describe the development of a CRISPRi platform in human RPE cells that would facilitate loss-of-function studies for AMD-associated genes. We report the generation of a human RPE cell line, ARPE19, that stably expresses dCas9-KRAB. This CRISPRi system utilises an inactive SpCas9 (dCas9) coupled with the transcriptional repressor domain Kruppel associated box (KRAB), which can be directed by targeting the sgRNA to induce specific gene repression [20]. We performed a transcriptomic analysis of human retina to prioritise AMD-associated genes for functional study and showed that *TMEM97* was highly expressed in human RPE/choroid. Using our CRISPRi RPE system, we showed that knockdown of *TMEM97* exerted a protective effect on ARPE19 against oxidative stresses. Our results support a functional role of *TMEM97* in regulating oxidative stress and degeneration of RPE, which have implications in the development of new therapeutic targets to improve treatment options for AMD.

## 2. Results

### 2.1. Generation of a Stable ARPE19 Cell Line for CRISPRi

**Figure 1:**
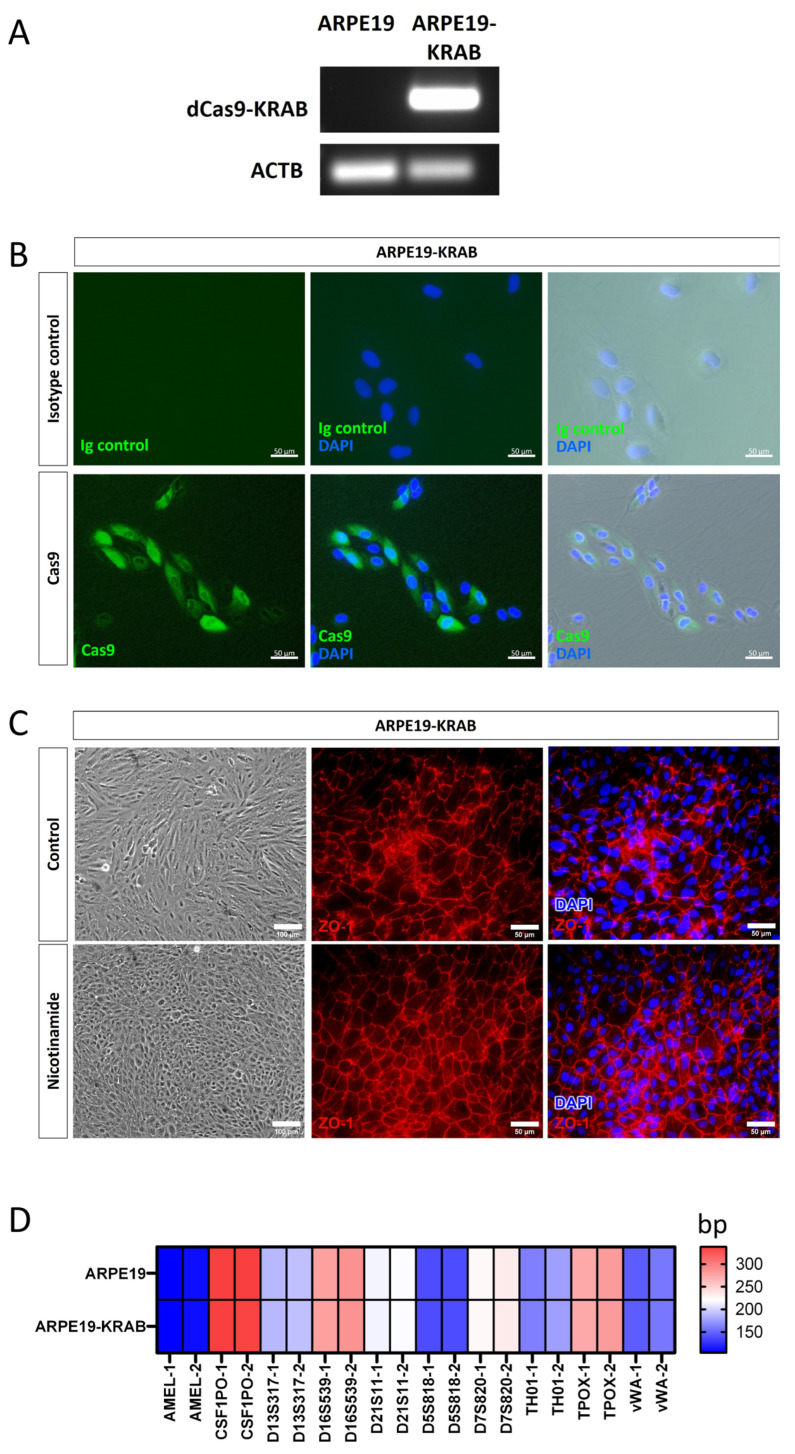
Generation of a stable CRISPRi human RPE cell line expressing dCas9-KRAB. Characterization of ARPE19-KRAB cell line showing expression of (**A**) KRAB by RT-PCR, and (**B**) SpCas9 by immunocytochemistry. (**C**) ARPE19-KRAB was able to form polarised monolayer upon nicotinamide treatment. Left panel shows representative morphology of ARPE19-KRAB treated with control condition (10% FCS) or differentiation media with nicotinamide. Middle and right panel show immunocytochemistry of tight junction protein ZO-1. (**D**) Heatmap of short tandem repeat analysis of 10 polymorphic markers. Alleles 1 and 2 are designated as −1 and −2 respectively.

To facilitate the use of CRISPRi in retinal research, we generated a human RPE cell line that stably expresses the transcriptional repressor dCas9-KRAB under the EF1α promoter, termed ARPE19-KRAB. We performed detailed characterisation of the derived ARPE19-KRAB cells. Our results showed that ARPE19-KRAB possessed stable expression of dCas9-KRAB for at least 2 months after transduction (Figure 1A). Furthermore, immunocytochemistry analysis showed that ARPE19-KRAB express detectable levels of SpCas9 protein (Figure 1B), while no signal for SpCas9 was detected in isotype control, nor in the parental ARPE19 (Figure 1B and Figure S1). Using an established method to induce polarisation of ARPE19 with nicotinamide treatment [21], we verified that ARPE19-KRAB retained the ability to form a monolayer with organised hexagonal RPE morphology, as shown by expression of the tight junction protein ZO-1 (Figure 1C). Finally, we performed short tandem repeat (STR) analysis and confirmed that ARPE19-KRAB retained the same STR signature of the parental ARPE19 cell line (Figure 1D). Together, these results validate the quality of the derived ARPE19-KRAB as a retinal pigment epithelium platform with stable expression of a CRISPRi transcriptional repressor.

### 2.2. Gene Expression of TMEM97/VTN Locus in Human Retina

**Figure 2:**
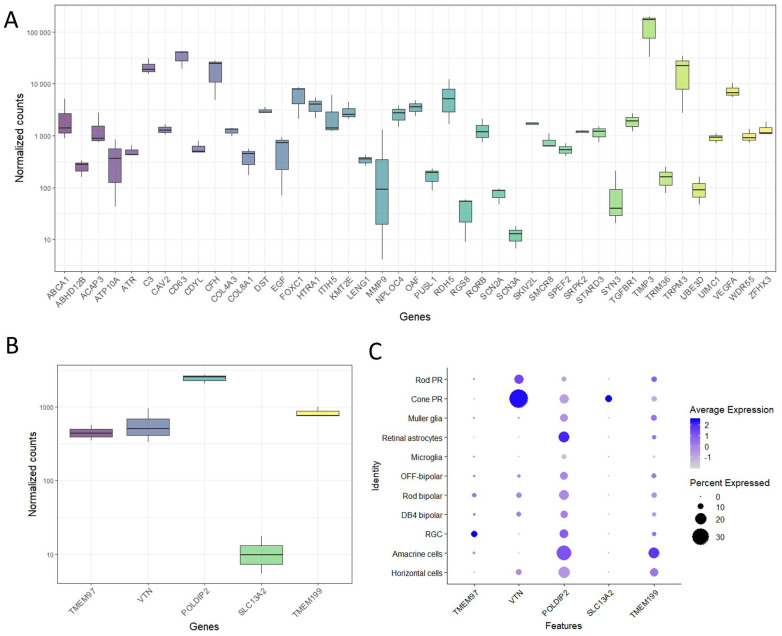
Expression of AMD-associated genes in human retina. (**A**) Bulk RNAseq results demonstrated expression of selected AMD-associated genes in human RPE/choroid (*n* = 3 donors). Expression levels of genes in the *TMEM97/VTN* locus in (**B**) human RPE/choroid as shown by bulk RNAseq, and (**C**) major retinal cells in human neural retina as shown by single cell-RNAseq (*n* = 3 donors; [22]). PR: photoreceptors; RGC; retinal ganglion cells.

To demonstrate the value of the ARPE19-KRAB system, we set out to pick an AMD-associated gene for further loss-of-function studies using CRISPRi. We performed RNA sequencing (RNAseq) in healthy human RPE/choroid samples from 3 donors ranging from 69 to 77 years old with short retrieval time (4–6 h, Supplementary Table S1). Using a list of AMD-associated genes previously identified by a large GWAS study [2], our transcriptomic data showed that many of the AMD-associated genes are expressed in human RPE/choroid (Figure 2A).

In particular, we focused on the AMD-associated locus *TMEM97*/*VTN*, which includes five genes: *TMEM97, VTN, POLDIP2, SLC13A2* and *TMEM199*. Our results showed that RPE/choroid express detectable levels of *TMEM97* (447 ± 35 Transcripts Per Million (TPM))*, VTN* (590 ± 155 TPM)*, POLDIP2* (2440 ± 16 TPM) and *TMEM199* (863 ± 154 TPM), but not *SLC13A2* (7 ± 5 TPM, Figure 2B). Next, we performed an in silico analysis using single cell RNAseq data we previously reported for adult human neural retina [22]. Our results revealed that the retinal ganglion cells possessed strong expression of *TMEM97* (Figure 2C). *POLDIP2* and *TMEM199* were expressed ubiquitously in many retinal cell types, including photoreceptors (rods and cones), Müller glia, astrocytes, bipolar cells, retinal ganglion cells, amacrine cells, and horizontal cells, with the exception that low/no expression was detected in microglia. On the other hand, *VTN* and *SLC13A2* were mainly expressed in the cone photoreceptors (Figure 2C). Collectively, these results show that most of the genes in *TMEM97/VTN* locus are expressed in both human neural retina and RPE. Given the predominant expression pattern in RPE and lack of ubiquitous expression pattern in neural retinal cells, we selected *TMEM97* as a candidate gene to perform further loss-of-function study using our ARPE19-KRAB system.

### 2.3. Loss-of-Function Study of TMEM97 Using CRISPRi

To induce knockdown of *TMEM97* expression, we designed three sgRNAs that target the proximity of the transcription start site (TSS) of *TMEM97* gene (Supplementary Figure S2) using the TSS defined by Ensembl (sgRNA 1), and two sgRNAs were designed using the TSS defined by the FANTOM/CAGE promoter dataset (sgRNA 2 and 3; Supplementary Table S2 [23]). We synthesized the three sgRNAs in an expression cassette driven by a U6 promoter, as we previously described [24]. To evaluate the efficiency of the three sgRNAs to knockdown *TMEM97* expression, we transiently co-transfected individual sgRNA together with a dCas9-KRAB plasmid into HEK293A cells using Lipofectamine 3000. qPCR analysis showed that sgRNA 1 and 3 did not induce knockdown of endogenous *TMEM97* levels in HEK293A, while sgRNA 2 achieved the highest knockdown level and was selected for further experiments (Supplementary Figure S3).

**Figure 3:**
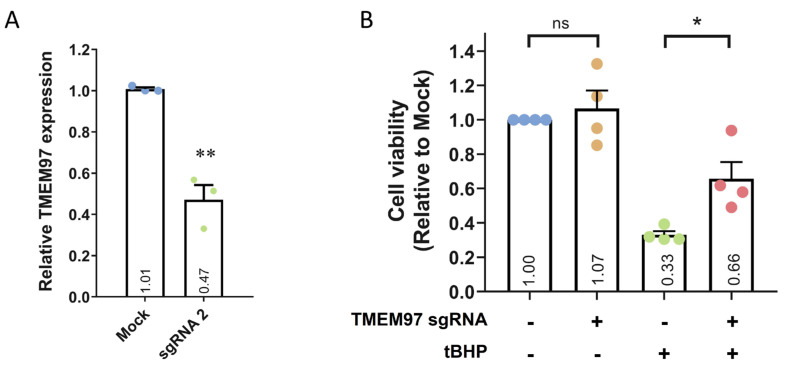
CRISPRi-mediated *TMEM97* knockdown and its effect on cell viability. (**A**) qPCR analysis of *TMEM97* knockdown using CRISPRi in ARPE19-KRAB. Results are presented as the mean ± SEM of three biological repeats, each with three technical repeats. ** indicates *p* < 0.01. (**B**) Cell viability analysis of ARPE19-KRAB following *TMEM97* knockdown in the presence or absence of tBHP. Results are presented as the mean ± SEM of four biological repeats, each with four to six technical repeats. ns indicates not significant; * indicates *p* < 0.05.

Next, we transfected sgRNA 2 into ARPE19-KRAB, which resulted in ~53% knockdown of *TMEM97* after 4 days (Figure 3A and Figure S4). Moreover, transfection with a control scrambled sgRNA did not result in *TMEM97* knockdown (Supplementary Figure S4). To further evaluate the specificity of this CRISPRi approach, we performed in silico analysis using Cas-OFFinder [25] to identify the off-targets of *TMEM97* sgRNA 2. No off-targets were identified for sgRNA 2 for alignments with up to 2 mismatches. When we adjusted the parameters to allow for three mismatches, five potential off-targets were identified, four of which were located in deep intronic regions, while one was not close to any known genes (Supplementary Table S3). Critically, no transcription start site was located within 1 kbp upstream and 1 kbp downstream of all 5 potential off-targets, supporting the specificity of our CRISPRi approach for *TMEM97* repression.

Using the ARPE19-KRAB system, we assessed if loss of *TMEM97* would affect cell viability of ARPE19. Our results demonstrated that CRISPRi-mediated knockdown of *TMEM97* did not affect the viability of ARPE19 cells (Figure 3B), suggesting that *TMEM97* is not critical in maintaining RPE cell viability under normal conditions. Given that oxidative stress plays a key role in RPE degeneration in AMD [26], we further treated ARPE19-KRAB with tert-Butyl hydroperoxide (tBHP) to induce oxidative stress and better mimic RPE under AMD pathological conditions. Cell viability analysis showed that tBHP treatment for 24 h resulted in dramatic cell death in ARPE19-KRAB (0.33 ± 0.02-fold compared to control, Figure 3B). Interestingly, *TMEM97* knockdown resulted in a significant increase in resistance to tBHP-induced cell death (0.66 ± 0.097-fold compared to control, *p* = 0.03).

**Figure 4:**
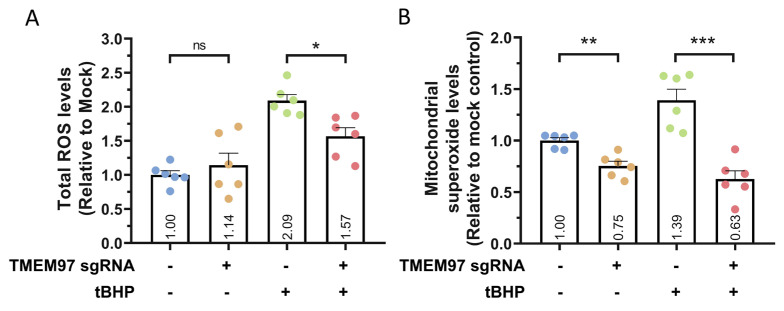
*TMEM97* knockdown resulted in reduced ROS levels. Quantification of (**A**) total ROS levels using CellRox assay and (**B**) mitochondrial superoxide levels using MitoSox assay, following *TMEM97* knockdown in ARPE19-KRAB in the presence or absence of tBHP. *n* = 6 repeats from 2 independent experiments, error bars represented SEM; ns indicates not significant; * indicates *p* < 0.05, ** indicates *p* < 0.01, *** indicates *p* < 0.001.

To further study the possible role of *TMEM97* in modulating oxidative stress, we utilised the CellRox assay to analyse the levels of reactive oxygen species (ROS). We first induced *TMEM97* knockdown in ARPE19-KRAB, followed by tBHP treatment for 24 h to induce oxidative stress. In the presence of tBHP, our results showed that *TMEM97* knockdown resulted in a significant decrease in tBHP-induced total ROS levels (*p* = 0.02, Figure 4A). Similarly, we utilised the MitoSox assay to analyse the mitochondrial superoxide levels. Following tBHP treatment for 24 h, our results indicated that *TMEM97* knockdown also reduced mitochondrial superoxide levels compared to control (*p* = 0.0002, Figure 4B). This provides further support for the protective effect of *TMEM97* knockdown against oxidative stress-mediated cell death. In the basal condition, we also observed a 25% decrease in mitochondrial superoxide levels following *TMEM97* knockdown (*p* = 0.001), but overall there were no statistically significant changes to the total ROS levels (*p* = 0.24, Figure 4). Together, these results show a novel role for *TMEM97* in regulating the oxidative stress and viability of RPE and demonstrate the potential of using CRISPRi for functional studies of AMD-associated genes in RPE cells.

## 3. Discussion

Functional validation of putative causal genes would foster a better understanding of AMD pathophysiology, which is important to develop better diagnosis and treatments for this disease. However, the study of AMD-associated genes in the retina using model organisms is challenging: the lack of macula in rodents represents a major limitation in modelling AMD, while primate studies are limited by the high research cost and the requirement of specialised housing facilities [27]. In this study, we present an in vitro human RPE model with CRISPRi for gene repression and demonstrate the value of our model for knockdown studies of AMD-associated genes using *TMEM97* as an example. We were able to identify a sgRNA for successful knockdown using gene annotation from the FANTOM/CAGE project, which is consistent with a previous study [28]. Using this system, we were able to design a sgRNA that targets the immediate downstream region of the TSS to knockdown the expression of *TMEM97*. To simplify our approach, the ARPE19-KRAB platform can be combined with a sgRNA expression cassette system [24] that can be commercially synthesized, providing a rapid approach to initiate gene knockdown studies using CRISPRi. For long-term studies, our ARPE19-KRAB platform could be complemented with inducible sgRNA expression, such as doxycycline or tamoxifen-inducible systems, to precisely control the duration of gene knockdown. In addition, the ARPE19-KRAB system can also be utilised for genome-wide screening using established CRISPRi libraries [18,19], which would enable future identification of genes that contribute to AMD pathophysiology, such as dysfunctional phagocytosis or degeneration of RPE. Collectively, this study provides a valuable tool to facilitate functional studies of genetic factors that contribute to RPE dysfunction in AMD.

Using the CRISPRi system, we showed that knockdown of *TMEM97* did not affect cell viability of ARPE19 under a normal physiological environment. On the other hand, in the presence of oxidative stress, *TMEM97* knockdown provided protective effects against cell death in ARPE19. Accumulation of oxidative stress is known to play a key role in AMD pathogenesis and progression, which can be caused by multiple risk factors including aging, light damage, and cigarette smoking [29]. Notably, a recent transcriptome-wide association study predicted that AMD cases exhibit a higher expression level of *TMEM97* compared with controls [30], suggesting that *TMEM97* levels may modulate RPE degeneration caused by oxidative stresses. Consistent with this, our results provide the first evidence that *TMEM97* plays an important role in regulating oxidative stress and cell survival of human RPE, supporting a potential role of *TMEM97* inhibition as a therapeutic strategy for treating AMD. Future gain-of-function studies for *TMEM97* would be important to further support its role in regulating oxidative stresses and cell death in RPE.

There are limitations in this study. Firstly, we used acute oxidative stress treatment to mimic the accumulated oxidative stress levels related to aging. However, other genetic variants and cellular stresses are also important in the pathogenesis of AMD, such as mitochondrial defects, lipofuscin accumulation, autophagy, and crosstalk between RPE and the immune and vascular systems [31]. Secondly, although the knockdown levels for *TMEM97* achieved by our CRISPRi approach were not very high, a clear phenotype in ARPE19 could be observed following *TMEM97* knockdown. Future studies to knockout *TMEM97* in human RPE models, for instance using CRISPR gene editing, would support our findings and would further elucidate its role in RPE biology and AMD pathophysiology. Finally, although ARPE19 is the most commonly used RPE cell line, which retains some RPE characteristics such as the ability to phagocytose photoreceptor outer segments [32], there are limitations for its use to model native RPE. For instance, ARPE19 display reduced levels of some RPE marker genes (e.g., CRALBP and RPE65) compared to native RPE [33], changes in morphology, heterogeneity, and karyotype following prolonged culture [21,34,35]. Therefore, the CRISPRi ARPE19 model reported here may not be suitable to study AMD genes with significantly different expression levels compared to native RPE. Other in vitro RPE models have been reported, including primary RPE and RPE derived from pluripotent stem cells (reviewed in [7,36]). Future research beyond this study using in vitro RPE models, such as primary human RPE or induced pluripotent stem cell-derived RPE [7], as well as in vivo animal models for AMD [37], would be important to confirm the role of *TMEM97* in regulating cell survival and oxidative stress in RPE. Further studies assessing the functional role of *TMEM97* in other retinal cells, such as photoreceptors, would also be of high interest. In addition, given the potential of CRISPRi for multiplexed gene repression [38], it would provide valuable complementary information to knockdown *TMEM97* together with other AMD-associated genes to determine if these genes have a synergistic contribution to retinal degeneration and the pathogenesis of AMD. In this regard, we provided a transcriptomic analysis of the human retina that can be used to prioritise AMD-associated genes, which could be further studied using our CRISPRi RPE platform.

Previous studies have demonstrated a role of the Sigma-2 receptor signalling in regulating apoptosis, cytotoxicity, and oxidative stress [9,10,11]. Our study extended these findings and showed that knockdown of *TMEM97* played a protective role in RPE against oxidative stresses, providing a potential drug target to alleviate RPE degeneration in AMD. Significant progress has been made to treat neurodegenerative diseases using Sigma-2 receptor inhibitors, including clinical trials in Alzheimer’s disease [39] and schizophrenia [40]. In particular, a Sigma-2 receptor antagonist CT1812 has demonstrated a safety profile in the clinic [39] and is currently being tested as a treatment for Alzheimer’s disease in a Phase II trial (ClinicalTrials.gov: NCT03507790). Future studies that evaluate the efficacy of Sigma-2 receptor antagonists in RPE could pave the way for the development of novel treatments to improve vision in patients with AMD.

In summary, this study presents an in vitro human RPE system with integrated CRISPRi for gene repression. We show that this in vitro CRISPRi system can be used for loss-of-function studies of AMD-associated genes. Using this CRISPRi system, we reveal a novel role of *TMEM97* in regulating human RPE viability against oxidative stress, providing supporting evidence for its role in AMD pathogenesis.

## 4. Methods

### 4.1. Donor Retina Collection

Collection of patient samples was approved by the Human Research Ethics committee of the Royal Victorian Eye and Ear Hospital (HREC13/1151H) and carried out in accordance with the approved guidelines. Informed consent was obtained from all donors, and the experiments conformed to the principles set out in the WMA Declaration of Helsinki. Post-mortem eye globes were collected by the Lions Eye Donation Service (Royal Victorian Eye and Ear Hospital, Melbourne, Australia) for donor cornea transplantation. The remaining eye globes were used to dissect the RPE/choroid layers for this study.

### 4.2. Transcriptome Analysis Using RNAseq

Total RNA was extracted using the RNeasy kit and treated with DNase 1 (Qiagen, Hilden, Germany). Three donor samples of human RPE/choroid were processed for RNAseq. RNAseq was performed by the Australian Genome Research Facility. Briefly, quality of the RNA samples was checked using a bioanalyzer, and transcriptome libraries were prepared using the TruSeq Stranded mRNA kit (Illumina, San Diego, CA, USA). Subsequently, samples were processed for 100 bp single-end sequencing using an Illumina Novaseq 6000 and yielded ~38–50 million reads per sample. The human reference transcriptome GRCh38 was used as an index, and transcript level quantification was performed using *Salmon* v1.0 to obtain gene-level counts [41]. The *tximport* package was used to import and summarize the gene-level counts, using the lengthScaledTPM function [42], and was imported to *DESeq2* for count normalisation and gene expression analysis [43]. Visualisation of gene expression was generated using the packages *ggplot2* [44]. The transcriptome data of the human RPE/choroid in this study are available in the NCBI Gene Expression Omnibus database (GSE181550), including raw data, processed data, information of the experimental design, sequencing, and processing pipeline.

### 4.3. Cell Culture

HEK293A, HEK293FT, ARPE19, and ARPE19-KRAB cells were cultured in DMEM (Thermo Fisher, Waltham, MA, USA) supplemented with 10% [*v*/*v*] Fetal Bovine Serum (FBS), 1× GlutaMAX, and 0.5% Penicillin/Streptomycin (all from Thermo Fisher). All cells were grown at 37 °C and 5% CO_2_ and passaged using 0.25% Trypsin-EDTA (Thermo Fisher, Waltham, MA, USA).

### 4.4. Lentivirus Generation

7 × 10^6^ HEK293FT cells were plated in a 10 cm dish one day prior to transfection and cultured with lentivirus packaging medium (Opti-MEM supplemented with 5% FBS and 200 µM Sodium pyruvate, all from Thermo Fisher). The lentiviruses were generated by co-transfecting the dCas9-KRAB plasmid (gifts from Kristen Brennand; #99372, Addgene, Watertown, MA, USA) with the three 3rd generation packaging vectors pMDLg/pRRE (gifts from Didier Trono, #12251, Addgene), pRSV-Rev (#12253, Addgene) pMD2.G (#12259, Addgene), using Lipofectamine 3000 (Thermo Fisher). The medium with lipofectamine 3000 was replaced with fresh media 6 h after transfection. Afterwards, the crude virus was collected at 48 and 72 h after transfection. The collected virus was filtered (0.45 µm filter, Sartorius, Göttingen, Germany) and concentrated using PEG-it overnight at 4 °C (System Biosciences, Palo Alto, CA, USA) and resuspended in cold PBS.

### 4.5. Generation of ARPE19-KRAB

ARPE19 cells were transduced with dCas9-KRAB lentiviruses overnight in the presence of 8 µg/mL of polybrene (Sigma-Aldrich, St. Louis, MO, USA), followed by selection with 2μg/mL puromycin (Sigma-Aldrich). We had previously performed a puromycin dosage study to determine that 2 μg/mL is the lowest puromycin concentration that can kill all ARPE19 cells. Following puromycin selection, all the transduced ARPE19 cells were further expanded to obtain the ARPE19-KRAB cell line. Polarisation induction of ARPE19-KRAB was performed as previously described [21]. On day 0, ARPE19-KRAB was cultured in MEMa media supplemented with 1% FBS, 0.5% Penicillin/Streptomycin (all from Thermo Fisher), 10 mM nicotinamide, 1% N1, 0.25 mg/mL taurine, 20 ng/mL hydrocortisone, and 0.013 ng/mL T3 (all from Sigma-Aldrich). On day 7, the sample was processed for immunocytochemistry and imaging analysis.

### 4.6. CRISPR Interference (CRISPRi)-Mediated Knockdown of Gene Expression

SpCas9 sgRNA expression cassettes containing a U6 promoter, sgRNA, and sgRNA scaffold were synthesized, amplified, and purified as previously described [24]. For HEK293A cells, on Day 0, 6 × 10^4^ cells were plated down in a well on a 12-well plate. On day 1, the cells were transiently transfected with 360 ng of sgRNA expression cassette and 800 ng of dCas9-KRAB using Lipofectamine 3000. For ARPE19-KRAB cells, on Day 0, 1 × 10^5^ cells were plated down in a well on a 12-well plate. On day 1, the cells were transiently transfected with 360 ng of sgRNA expression cassette using RNAimax (Thermo Fisher). For both HEK293A and ARPE19-KRAB cells, a mock control (no DNA transfected) was utilised as a negative control in every individual experiment. On Day 4, both HEK293A and ARPE19-KRAB samples were processed for RNA extraction and qPCR analysis to assess gene expression levels.

### 4.7. PCR and qPCR Analysis

Total RNA was extracted using the Illustra RNAspin kit and treated with DNase 1 (GE Healthcare, Chicago, IL, USA). cDNA synthesis was performed using a high-capacity cDNA reverse transcription kit with RNase inhibitor (Thermo Fisher). For PCR analysis, a 25 µL PCR reaction was set up using the KOD hot start DNA polymerase (Merck, Rahway, NJ, USA), following manufacturer’s instructions. The following primers were used: KRAB (FWD: CGTGAGGAGTGGAAATTGCTGG, REV: TTCCCCCTTTTCGAGCCTAAGG) and *ACTB* (FWD: CCCTGGCACCCAGCAC, REV: GCCGATCCACACGGAGTAC). The thermal profile used was 95 °C for 2 min; 30 cycles of 95 °C for 20 s, annealing temp for 20 s (KRAB: 65.8 °C, *ACTB*: 73.9 °C), 70 °C for 15 s; followed by 70 °C for 5 min. The PCR product was visualised in a 1% agarose gel. For qPCR, TaqMan gene expression assays were performed using TaqMan Fast Advanced Master Mix (Thermo Fisher) as previously described [45,46], using the following TaqMan probes for *TMEM97* (Hs00299877_m1) and the housekeeping gene β-actin (Hs99999903_m1). The TaqMan assay was performed using the ABI 7500 or StepOne plus qPCR machine, following manufacturer’s instructions (Thermo Fisher). The delta delta Ct method was used to calculate and compare relative mRNA levels to control.

### 4.8. Immunoblotting

Western blot analysis was performed following conventional procedures. Briefly, cell pellets were resuspended in three volumes of 1× RIPA buffer and sonicated. The lysate was mixed with 4x Laemmli Sample Buffer (Bio-Rad, Hercules, CA, USA) and 2-Mercaptoethanol (1:40, Sigma-Aldrich) and heated at 95 °C for 5 min. The lysate was loaded on the 4–15% Mini-PROTEAN TGX Precast Protein Gels (Bio-Rad) for separation and transferred to the PVDF membrane. The blots are blocked and incubated with rabbit anti-*TMEM97* antibody (1:400, #OAAB22200, Aviva Systems Biology, San Diego, CA, USA) as primary antibody overnight at 4 °C. After washing, the membranes were incubated with a secondary HRP conjugated anti-rabbit IgG antibody (1:2500, GE Healthcare) and developed by chemiluminescence using ECL reagents according to the manufacturer’s instructions (Thermo Fisher).

### 4.9. Immunocytochemistry

Standard immunocytochemistry procedures were carried out, as we previously described [47]. Briefly, samples were fixed in 4% paraformaldehyde, followed by blocking with 10% serum (Sigma-Aldrich) and permeabilization with 0.1% Triton X-100 (Sigma-Aldrich). The samples were then immunostained with antibodies against Cas9 (#14697, Cell Signaling, Danvers, United States) or ZO-1 (#339100, Thermo Fisher), Alexa Fluor 488 secondary antibodies (Abcam, Cambridge, UK), and nuclear counterstain with DAPI (Sigma-Aldrich). Samples were imaged using a Zeiss Axio Vert.A1 fluorescent microscope. The specificity of the immunostaining was confirmed using isotype control antibodies from the appropriate species.

### 4.10. Cell Viability Assay

Cell viability assay was performed using a CellTiter-Glo Luminescent Cell Viability Assay (#G9241, Promega, Madison, WI, USA) following the manufacturer’s instructions. Briefly, 4 × 10^4^ cells were seeded in a well of a 96-well plate on Day 0, followed by CRISPRi on Day 1. For some conditions, the cells were treated with 100 mM tert-Butyl Hydroperoxide (tBHP) on Day 3. On Day 4, old media was replaced with 25 μL fresh media in each well, followed by adding 25 μL CellTiter-Glo reagent before incubating at room temperature for 10 min to initiate a luminescent reaction. A volume of 40 μL of cell lysate from each well was loaded to an opaque white luminometer plate to measure luminescence using a Spark 20 M microplate reader (Tecan). The OD reading was normalised to the control condition without sgRNA and tBHP treatment, and the results are presented as fold change relative to the control condition.

### 4.11. Reactive Oxygen Species (ROS) Analysis

Mitochondrial superoxide and total cellular ROS levels were assessed using MitoSOX and CellROX dyes respectively (All from Thermo Fisher). Following 24 h of treatment with tBHP, cells were stained with 5 µM MitoSOX for 20 min or 5 µM CellROX for 30 min in DMEM low glucose (Thermo Fisher) at 37 °C in a humidified CO_2_ incubator. Cells were then washed twice with HBSS++ (Hank’s Balanced Salt Solution; Sigma-Aldrich) and imaged immediately in warm HBSS++ solution using an Olympus IX-71 microscope. Images were captured at 200 times magnification and the fluorescence intensities were analysed using ImageJ software. A total of 80 cells per group for each condition were analysed per replicate. The final fluorescence intensity was normalised to the mock control and expressed as fold change relative to mock.

### 4.12. Short Tandem Repeat Analysis

Genomic DNA of ARPE19 and ARPE19-KRAB were extracted using the Wizard SV Genomic DNA Purification kit (Promega), following manufacturer’s instructions. Short tandem repeat analysis was performed using the GenePrint 10 system (Promega) by the Australian Genome Research Facility.

### 4.13. Statistical Analysis

Statistical analysis was performed on biological repeats using paired *t*-test for assessment of *TMEM97* knockdown levels, cell viability, total ROS levels, and mitochondrial superoxide levels (Graphpad Prism). *p* < 0.05 was used to establish statistical significance.

## Data Availability

The transcriptome data of the human RPE/choroid in this study are available in the NCBI Gene Expression Omnibus database (GSE181550).

## Supplementary Materials

The following supporting information can be downloaded at: https://www.mdpi.com/article/10.3390/ijms24043417/s1.
