# Supplementary material for: Development of a CRISPRi Human Retinal Pigmented Epithelium Model for Functional Study of Age-Related Macular Degeneration Genes
Source: Int J Mol Sci. 2023 Feb 8;24(4):3417. doi: 10.3390/ijms24043417 (PMC9962760; doi:10.3390/ijms24043417)
Supplement: Supplementary file 1 [file ijms-24-03417-s001.zip › ijms-2187434-supplementary.pdf]

Supplementary information

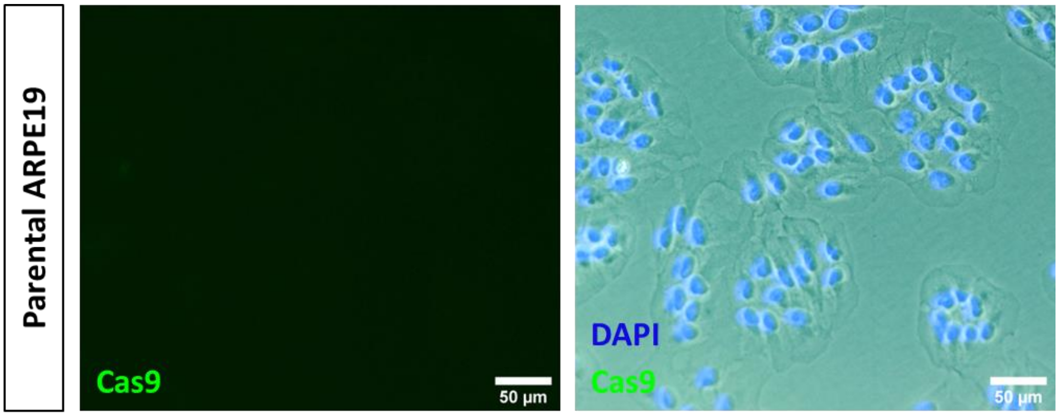

Figure S1. Immunostaining of SpCas9 in parental cell line ARPE19 confirmed absence of SpCas9 signal.

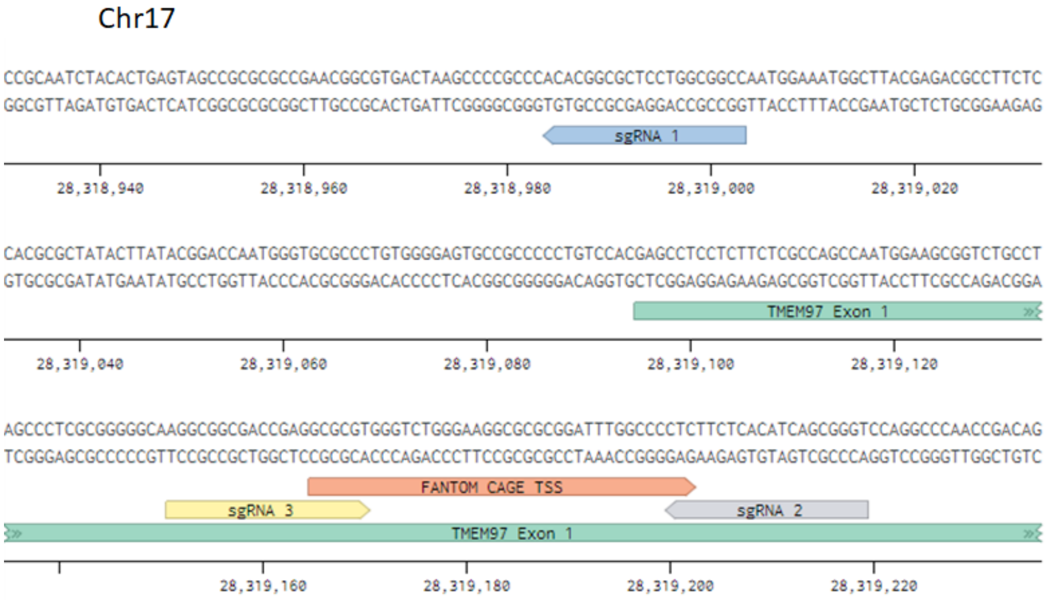

Figure S2. Schematic diagram of the 5′ region of human *TMEM97* gene with sgRNA target areas near the transcription start site (TSS). The green region marks exon 1 of *TMEM97* gene as determined by Ensembl. The orange region marks the TSS as determined by FANTOM CAGE data. Target sites for sgRNA are marked in blue (sgRNA 1), grey (sgRNA 2) and yellow (sgRNA 3).

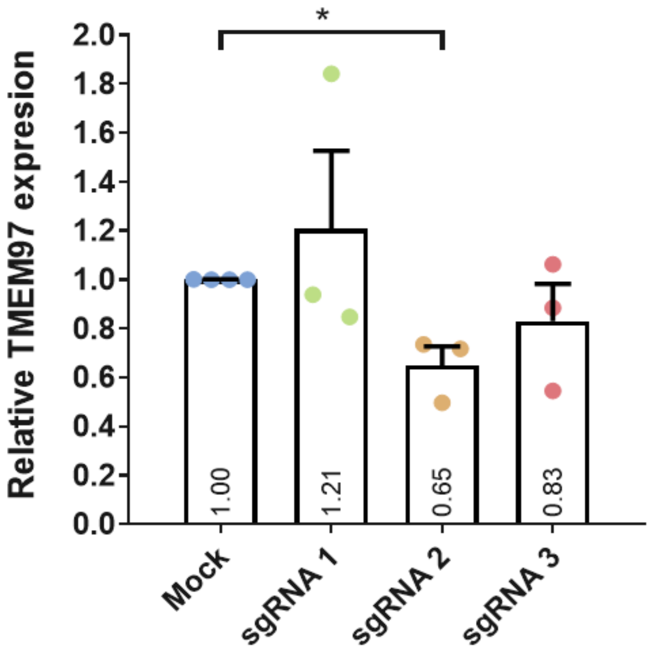

Figure S3. qPCR analysis of CRISPRi knockdown of *TMEM97* expression in HEK293A using 3 different sgRNA. Results represented as the mean ± SEM of three biological repeats, each with three technical repeats. * indicates p<0.05.

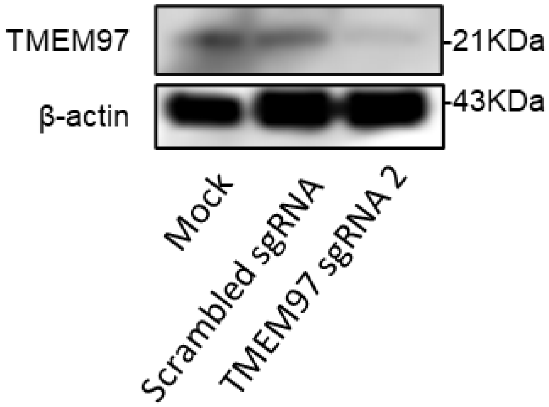

Figure S4. Westernblot analysis of TMEM97 expression in ARPE19-KRAB transfected with TMEM97 sgRNA 2 or scrambled sgRNA as a negative control.

Table S1. Details for donor RPE samples used for RNAseq.

Retina	Donor ID	Eye bulb	Age (yrs)	Sex	Retrieval time (hrs)	Ocular history
1	17-080	Right	77	F	4 hrs	Healthy
2	17-172	Left	69	F	4 hrs	Healthy
3	18-011	Left	71	M	6 hrs	Bilateral intraocular lens, exophthalmos

Table S2. Information of sgRNAs used in this study. TSS distance is based on the transcription start site defined by Ensembl. On-target score is based on [23].

Name	TSS distance	Strand	Sequence	PAM	Design	On-target score
TMEM97 sgRNA 1	-91bp	-	GGCCGCCAGGAGCGCCGTGT	GGG	Ensembl	50.6
TMEM97 sgRNA 2	+105bp	-	ACCCGCTGATGTGAGAAGAG	GGG	FANTOM CAGE	65.9
TMEM97 sgRNA 3	+56bp	+	AGGCGGCGACCGAGGCGCGT	GGG	FANTOM CAGE	56

Table S3. Off-targets alignment of *TMEM97* sgRNA2 using CasOFFinder with up to 3 mismatches. TSS: transcription start site.

Target	Chromosome	Position	Direction	Mismatch	Genomic location	TSS within 1kbp upstream and 1kbp downstream
crRNA: ACCCGCTGATGTGAGAAGAGNGG<br>DNA: ACCCGCTGATGTGAGAAGAGGGG	chr17	28319196	-	0	TMEM97 exon 1	TMEM97
crRNA: ACCCGCTGATGTGAGAAGAGNGG<br>DNA: ACatGCTGATGTGAGAtGAGTGG	chr8	17732170	+	3	N/A	N/A
crRNA: ACCCGCTGATGTGAGAAGAGNGG<br>DNA: gCCCGCTttTGTGAGAAGAGCGG	chr5	157491565	-	3	Intronic region of ADAM19	N/A<br>N/A
crRNA: ACCCGCTGATGTGAGAAGAGNGG<br>DNA: gCCCtCTcATGTGAGAAGAGAGG	chr14	71953121	+	3	Intronic region of RGS6	N/A
crRNA: ACCCGCTGATGTGAGAAGAGNGG<br>DNA: ACCCGCTGATGTtAGAAGctGGG	chr10	70942247	+	3	Intronic region of LINC02622	N/A

crRNA: ACCCGCTGATGTGAGAAGAGNGG DNA: ACCCGCTGtgGTGAGAAGtGAGG	chr13	93671677	-	3	Intronic region of GPC6	N/A
